# Triple guidance of choledochoscopy, ultrasonography, and computed tomography facilitates percutaneous catheter drainage of infected walled-off necrosis

**DOI:** 10.1186/s13244-021-01087-2

**Published:** 2021-09-27

**Authors:** Hui Zhang, Xu-dong Wen, Xiao Ma, Yong-qiang Zhu, Zhi-wei Jiang, Shang-qing Huang, Tao Wang, Wei-hui Liu

**Affiliations:** 1 General Surgery Center, The General Hospital of Western Theater Command (Chengdu Military General Hospital), Chengdu, 610083 Sichuan Province China; 2 Department of Gastroenterology and Hepatology, Chengdu First People’s Hospital, Chengdu, 610016 Sichuan Province China; 3 Department of Gastroenterology and Hepatology, Sichuan Provincial People’s Hospital, University of Electronic Science and Technology of China, Chengdu, 610072 Sichuan Province China

**Keywords:** Choledochoscope, Imaging fusion, Percutaneous catheter drainage, Walled-off necrosis

## Abstract

**Objectives:**

Percutaneous catheter drainage (PCD) is usually performed to treat acute pancreatitis complicated by infected walled-off necrosis (WON). Insufficient drainage of infected WON may lead to a prolonged recovery process. Here, we introduce a modified PCD strategy that uses the triple guidance of choledochoscopy, ultrasonography, and computed tomography (CUC-PCD) to improve the therapeutic efficiency.

**Methods:**

This study retrospectively analysed 73 patients with acute pancreatitis-related WON from January 2015 to January 2021. The first 38 patients were treated by ultrasonography/computed tomography-guided PCD (UC-PCD), and the next consecutive 35 patients by CUC-PCD. Perioperative data, procedural technical information, treatment outcomes, and follow-up data were collected.

**Results:**

Demographic characteristics were statistically comparable between the two treatment groups (*p* > 0.05). After 48 h of PCD treatment, the CUC-PCD group achieved a significantly smaller size of the infected WON (*p* = 0.023), lower inflammatory response indexes (*p* = 0.020 for white blood cells, and *p* = 0.031 for C-reactive protein), and severity scores than the UC-PCD group (*p* < 0.05). Less catheter duration (*p* = 0.001), hospitalisation duration (*p* = 0.000), and global costs (*p* = 0.000) were observed in the CUC-PCD group compared to the UC-PCD group. There were no differences between the two groups regarding the rate of complications.

**Conclusions:**

CUC-PCD is a safe and efficient approach with potential clinical applicability for treating infected WON owing to its feasibility in placing the drainage catheter at the optimal location in real time and performing primary necrosectomy without sinus tract formation and enlargement.

## Key points

- Efficient drainage in PCD requires that the catheter is placed at the sloping part of the necrotic cavity.
- Choledochoscope-assisted US/CT image-fused guidance facilitates the catheter placement during the PCD.
- Faster volume reduction, lower inflammatory response indexes and severity scores, and shorter catheter duration were observed by CUC-PCD.

## Introduction

Acute pancreatitis (AP) is a common disease with multiple complications [1]. For local complications, the Atlanta consensus demonstrated that the presence or absence of pancreatic/peripancreatic infected fluid collections was the key determinant of outcome [2]. It also has been verified that AP patients with necrotic infection were more likely to develop potentially fatal complications [3, 4]. The accumulation of peripancreatic necrotizing fluid 4 weeks after the onset of pancreatitis is defined as walled-off necrosis (WON) [5]. Due to the location in deep anatomical planes, and the potential risk of intestinal fistula, regional portal hypertension, upper gastrointestinal haemorrhage, etc., the treatment of WON is considered challenging and adds to the difficulties faced by the patients [6–8].

**Fig. 1:**
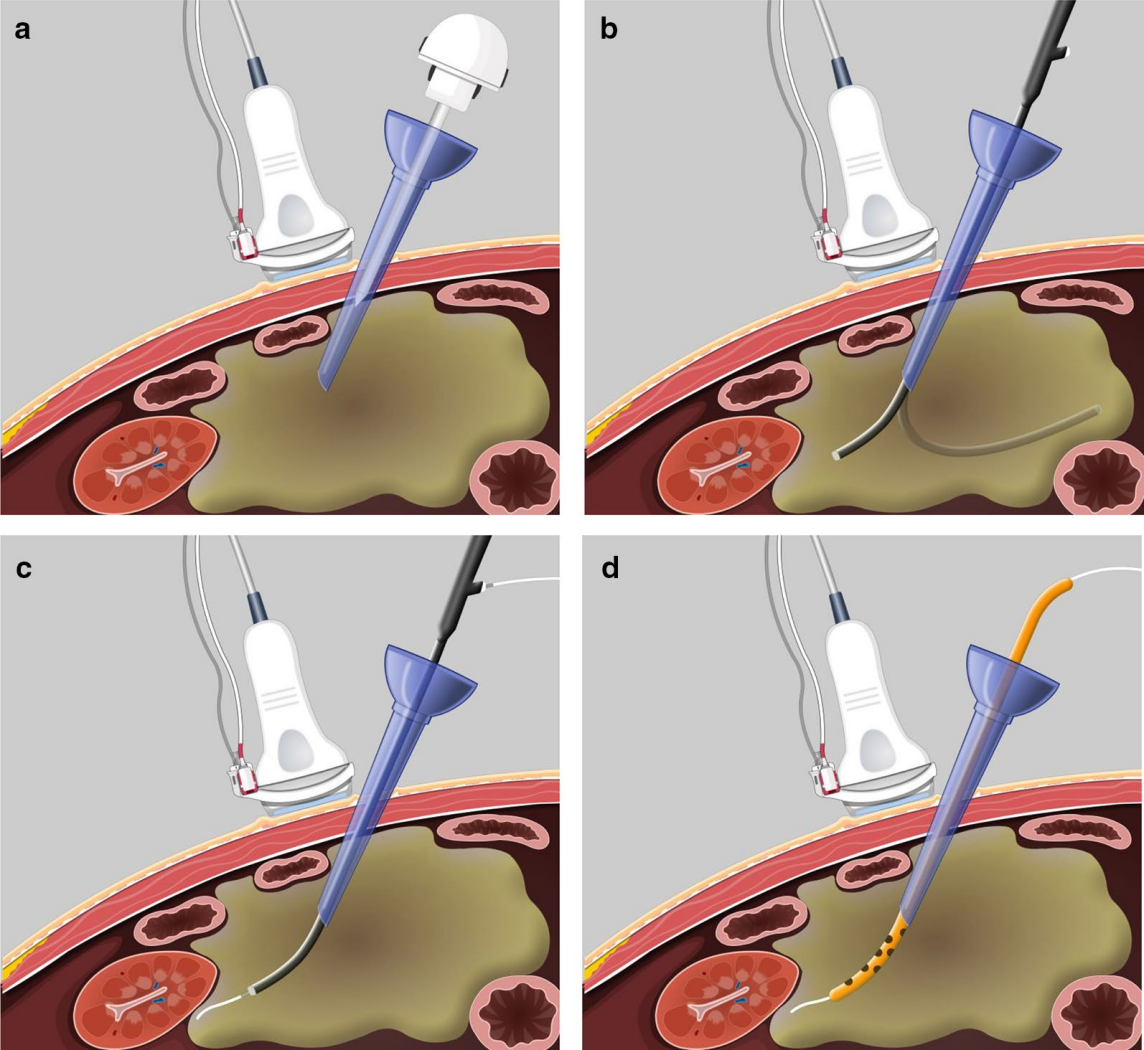
Schematic of the modified percutaneous catheter drainage procedure under the triple guidance of choledochoscopy, ultrasonography (US), and computed tomography (CT). **a** A laparoscopic trocar is inserted into the necrotic cavity under the guidance of an US/CT imaging system. **b** After the withdrawal of the core, a choledochoscope is inserted through the trocar to detect the necrotic cavity and to debride the necrosis if needed. **c** Under the triple-guidance system, the guidewire is placed at the sloping position of the walled-off necrosis (WON), penetrating the necrotic cavity. **d** A multi-side-hole catheter is then inserted along the guidewire to drain the WON

In patients with infected WON, efficient percutaneous catheter drainage (PCD) has been reported to be associated with favourable clinical outcomes [9]. However, it was observed clinically that if an improper puncture route was selected, the infected WON did not adequately respond to PCD [10, 11]. To overcome this insufficiency, we verified previously that ultrasonography (US)/computer tomography (CT) image-fused guidance was reliable for ensuring a safe puncture route by accurately visualising the WON’s location, scope, and quantity of infected fluid as well as displaying the relationship with the neighbouring organs [12]. However, the selected safe puncture route generally could not simultaneously ensure sufficient drainage by verifying that the drainage catheter penetrates the necrotic cavity and reaches the sloping position [6]. Based on US/CT-guided PCD (UC-PCD), we used a specific laparoscopic trocar as a puncture needle to establish a pathway to reach the WON, following which instant cholangioscopic interventions facilitated the PCD process through the fixed trocar channel. By choledochoscope/US/CT-guided PCD (CUC-PCD), the catheter could be placed at the optimum position in the necrotic cavity to improve the drainage efficacy (Fig. 1). The aim of this retrospective study was to investigate the efficiency and safety of CUC-PCD for the treatment of infected WON.

## Methods

### Patients

The study included 73 patients with mild-severe acute pancreatitis (MSAP) and severe acute pancreatitis (SAP) that were admitted to our hospital from January 2015 to January 2021. All patients developed WON with accompanying infection four weeks after the onset of MSAP/SAP and subsequently underwent PCD. MSAP, SAP, and WON were diagnosed on the basis of the revised standards of Atlanta [2]. The first 38 patients included from January 2015 to June 2018 underwent UC-PCD, whereas the next 35 patients included from June 2018 to January 2021 underwent CUC-PCD, as we gradually observed the efficacy of the choledochoscope for drainage tube placement in the process of UC-PCD. This study was performed in accordance with clinical study protocols and the principles of the Declaration of Helsinki (modified 2000) and was approved by the Research Care and Ethics Committee at our institution (No. SPPHCT2021–0012). Informed consent for the interventional procedures was obtained from all patients or their families. Data were collected and analysed retrospectively.

### Inclusion and exclusion criteria

#### Inclusion criteria

(1) Adults (> 18 years old) who experienced their first episode of MSAP or SAP, and underwent PCD. (2) Only one WON (encapsulated aggregation) diagnosed according to abdominal CT images. (3) WON accompanied by infection (diagnostic basis: excessive leukocytes in routine blood examination, fever, and positive bacterial culture of the drainage liquid obtained by fine needle aspiration).

#### Exclusion criteria

(1) Patients with WON whose CT images showed no viable percutaneous puncture route for PCD. (2) Patients with independent and multiple WONs, needing several drainages. (3) Patients with autoimmune deficiency, suspected malignancy of the pancreas or biliary tree, or previous abdominal operation.

### Technical procedures

#### Therapeutic equipment and materials

GE LOGIQ E9 diasonograph (from 3.5 to 5.0 MHz) with C1-5 probe (GE, USA), magnetic positioner and spare parts (GM, USA), disposable laparoscopic puncture trocar (JL5MN, Youjun Care, China), electronic choledochoscope (CHF-P60, Olympus, Japan), hydrophilic drainage catheter (Neo-Hydro, Bioteque Corp., Taiwan), T-tube (Zhanjiang Star Enterprise Co., Ltd., China), and guide wire (MTN-BM-89/45-A, Micro-tech Nanjing, China) were used.

#### PCD intervention

Location and size of the WON were assessed by two radiologists (> 5 years and 1000 times of CT-mediated abdominal punctures) by reviewing the imaging results. The PCD procedures were performed by attending surgeons with consistent technique and were guided by US doctors (> 5 years and 3000 times of US-mediated abdominal punctures).

##### Catheter placement by imaging fusion in UC-PCD

Patients in the UC-PCD group underwent real-time imaging fusion to determine the puncture pathway and drainage placement. Seldinger technique was performed by placement of a pig tail drainage catheter (8 Fr) along the route in the sloping position of the WON.

##### Tunnel establishment

**Fig. 2:**
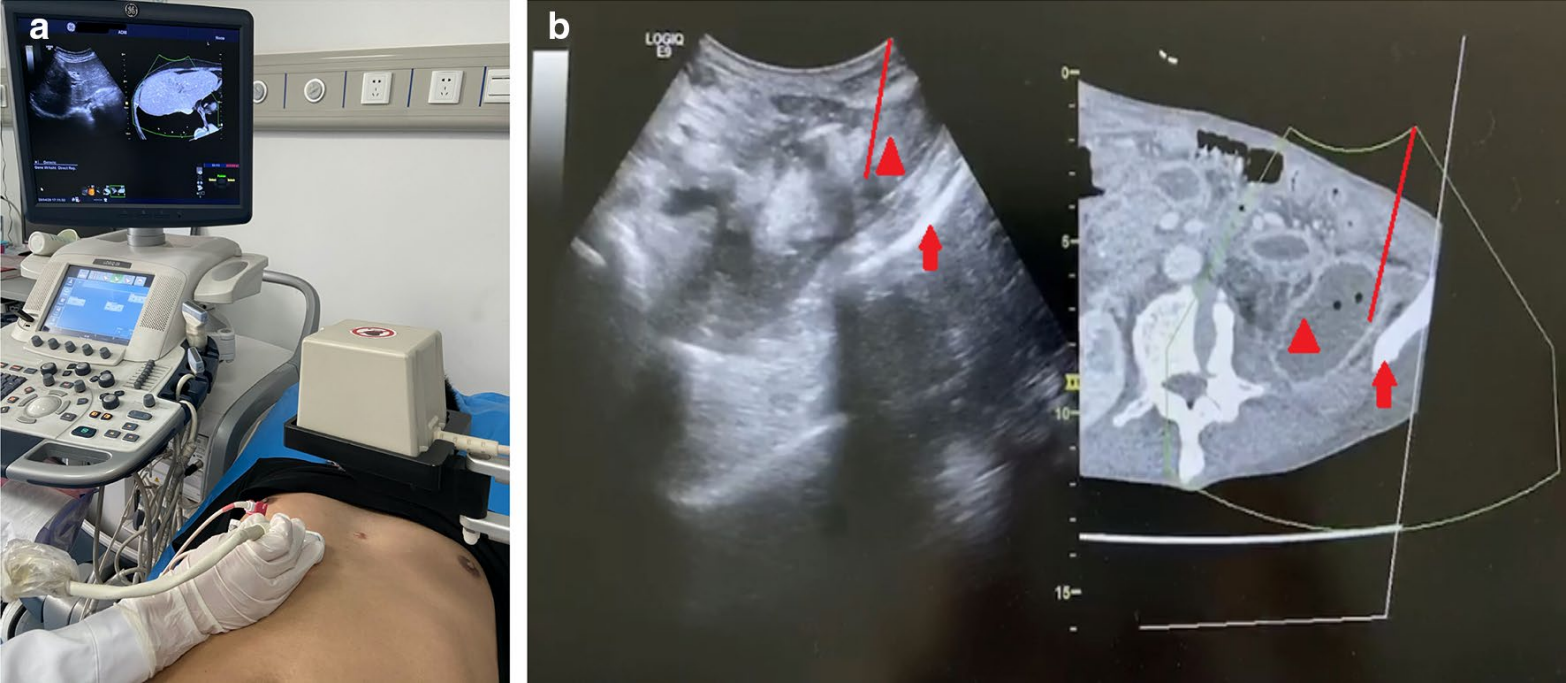
Selection of the puncture pathway under the guidance of an ultrasonography (US)/computer tomography (CT) imaging fusion system. **a** The US/CT imaging fusion system consists of a magnetic sensor-loaded ultrasonic detector, magnetic generator, and magnetic receiver, and the images of the liver are used for initial calibration. **b** The integrated image is displayed on the screen, puncture pathway (left midaxillary line) is indicated by red lines, walled-off necrosis (retroperitoneal area behind the colon) is indicated by red triangles, and the left ilium is indicated by red arrows

According to previous reports, the ultrasound/CT image fusion-guided puncture procedures are briefly described as follows [12]: The CT images (DICOM format) for fusion were imported into the diasonograph to obtain the imaging data. Then, the popular in vivo visualisation method was used to offer a real-time display of the corresponding plane, using an US probe for scanning (Fig. 2a). The WON position, scope, and surrounding important organs were identified in the US and CT images (Fig. 2b). The trocar puncture point was determined, and a safe puncture pathway was displayed to facilitate the insertion of the choledochoscope into the WON. After marking the skin surface and administering local anaesthesia, the trocar was placed according to the quasi-puncture path to access the necrotic space under US guidance.

##### Catheter placement by choledochoscopy in CUC-PCD

**Fig. 3:**
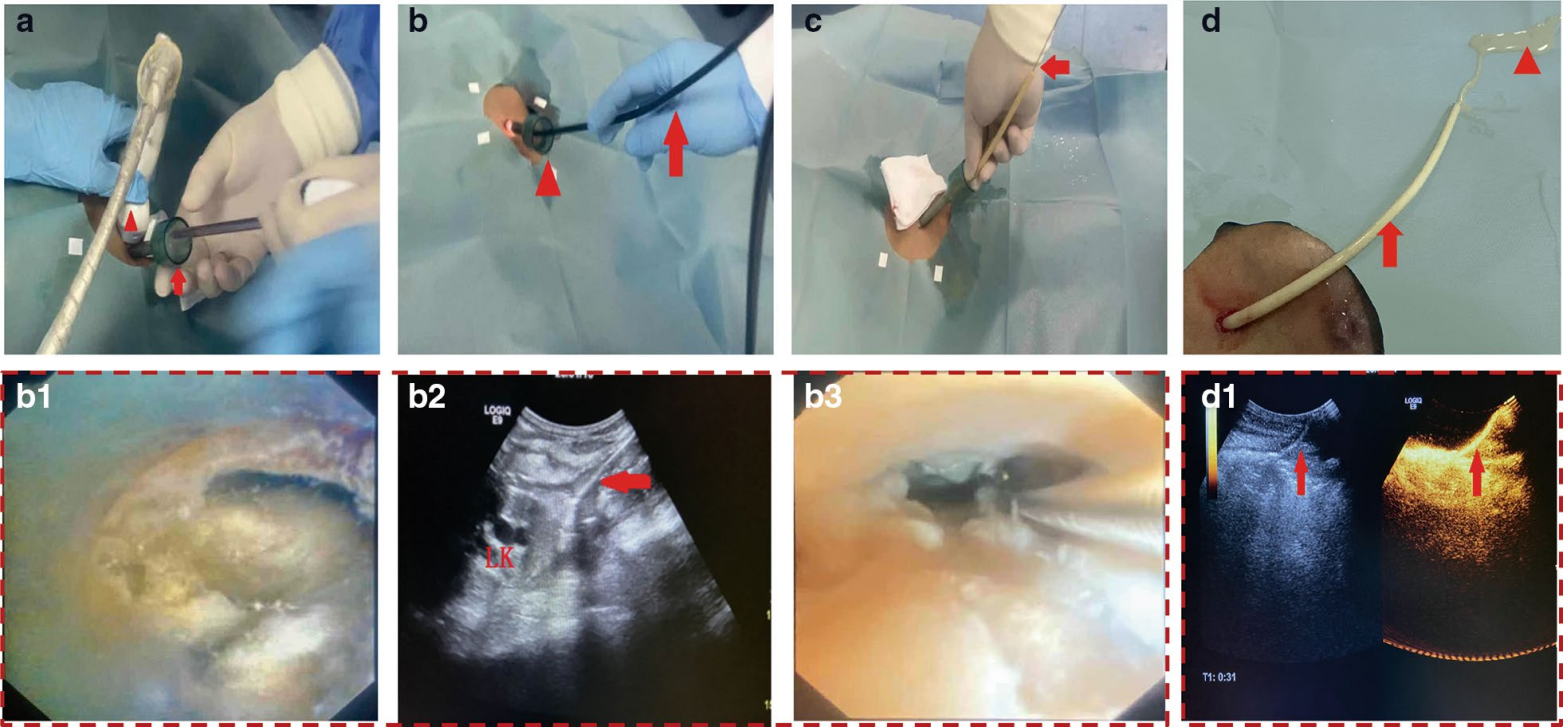
Images of modified percutaneous catheter drainage under the triple guidance of choledochoscopy, ultrasonography, and computed tomography. **a** After puncture by the trocar (red arrow), the core is withdrawn to establish a channel for the choledochoscope. **b** The cholangioscope (red arrow) is introduced through the trocar (red triangle), and the condition of the inner walled-off necrosis (WON) is observed by the choledochoscope (**b1**) and ultrasonography (**b2**, red arrow indicates the choledochoscope) in real time, followed by introduction of a guide wire to target the lowest part of the WON (**b3**). **c** A multi-side-hole catheter (red arrows, 16 Fr) is inserted along the guidewire to fully drain the WON, and abscess accumulation is observed by contrast-enhanced ultrasonography. **d** The liquefied necrotic tissue (red triangle) is successfully drained using the catheter (red arrow) and verified by contrast-enhanced ultrasonography (**d1**)

The core of the trocar was slowly withdrawn (Fig. 3a), followed by choledochoscope insertion through the fixed trocar (Fig. 3b). Necrotic materials could be visualised by the choledochoscope (Fig. 3b1), which was guided by US/CT (Fig. 3b2) to reach the sloping position in the necrotic cavity. Then, a guidewire was introduced (Fig. 3b3), and a home-made multi-side-hole drainage catheter (16 Fr) was placed along the guidewire to the choledochoscope-guided position (Fig. 3c). To confirm the configuration and drainage range of the drainage catheter, a US contrast agent (SonoVue, Bracco Diagnostics Inc., Sweden) was injected into the catheter and visualised by means of contrast-enhanced US. Finally, the unobstructed catheter was used to drain the liquid abscess (Fig. 3d).

#### Catheter expansion and necrosectomy

When insufficient drainage of the liquefied necrotic tissue was observed 7 days after the initial intervention, patients underwent catheter expansion to dilatate the sinus tract under US guidance. In the UC-PCD group, the 8 Fr drainage tube was expanded to 16–24 Fr; while in CUC-PCD group, the 16 Fr T-tube was expanded to 20–24 Fr. The visualised necrotic materials were removed by a combination of cholangioscopic interventions, including intermittent lavage as well as extraction by basket and biopsy clamp.

#### Indicators for removal of the drainage catheter

The drainage tube was removed when all of the following conditions were satisfied: (1) absence of fever for three consecutive days; (2) no abdominal pain and a normal white blood cell count; (3) the necrotic cavity had shrunk to 2 cm or disappeared on a serial CT scan/US; and (4) less than 10 ml of fluid was drained from the abscess for 3 consecutive days.

### Observation indicators

Observation indicators included the demographic indexes (sex, age) of the patients, aetiology, classification of AP, location of the WON, relevant laboratory inflammatory indexes (white blood cell [WBC] count, and C-reactive protein [CRP] level before and after PCD intervention), severity scores (acute physiology and chronic health evaluation [APACHE] II, computed tomography severity index [CTSI], and Ranson), necrotic fluids volume before and 48 h after the PCD procedure, operation indexes of PCD (including time from the onset to the first PCD, puncture area, diameter of the drainage tubes used, necrosectomy time), complications of PCD intervention and infection of AP, hospitalisation, and treatment costs. The size of the WON was measured by virtual organ computer-aided analysis (VOCAL) using three-dimensional US [13]. Effective imaging was defined as a decrease in the WON size, measured by VOCAL, by more than 60% after the PCD intervention compared to the size at the onset.

### Statistical analyses

All statistical analyses were performed using SPSS 19.0 (IBM SPSS, USA). All data are presented as percentages or means ± standard deviation. Statistical comparisons were done by using the *t* test or Wilcoxon rank sum test for continuous variables, and the chi-square test or Fisher’s exact test for categorical variables. A *p* value < 0.05 was defined as significant.

## Results

### Demographic characteristics

Age (*p* = 0.432), sex (*p* = 0.872), aetiology (*p* = 0.898), classification of AP (*p* = 0.415), location and size of WON (*p* = 0.543), biochemical parameters (WBC [*p* = 0.753], and CRP [*p* = 0.403]), and severity ratings (APACHE II [*p* = 0.599], Ranson [*p* = 0.110], and CTSI [*p* = 0.813]) were similar between the UC-PCD and CUC-PCD groups (Table 1). In both groups, gallstones were the most common cause of AP, and 49.31% of the patients were diagnosed with SAP, while 50.68% were diagnosed with MSAP. The location of the WON was anterior or above the pancreatic region in 12.33% patients, left anterior renal space in 53.42%, and right anterior renal space in 34.25% patients. The average size of the WON was 144.1 ± 20.8 cm^3^ and 156.2 ± 33.1 cm^3^ in the UC-PCD group and CUC-PCD group, respectively. Regarding biochemical parameters, WBC (16.2 ± 2.6 × 10^9^/L and 16.9 ± 2.8 × 10^9^/L) and CRP (126.3 ± 36.4 mg/L and 133.3 ± 34.7 mg/L) were relatively high in the UC-PCD and CUC-PCD groups, respectively.

**Table 1:** Basal characteristics of 73 infected WON patients enrolled in this study

Variable	UC-PCD (*n* = 38)	CUC-PCD (*n* = 35)	*p* value
Age (years)	46.2 ± 12.2	44.0 ± 11.6	0.432
Gender (*n* [%])			
Male	21 [55]	20 [57]	0.872
Female	17 [45]	15 [43]	
Aetiology (*n* [%])			0.898
Alcohol	8 [21]	10 [29]	
Gallstones	23 [61]	19 [54]	
Idiopathic	5 [13]	4 [11]	
Others	2 [5]	2 [6]	
Classification of AP (*n* [%])			0.415
Severe	17 [45]	19 [54]	
Moderately severe	21 [55]	16 [46]	
Mild	0	0	
Location of WON (*n* [%])			0.543
Anterior or above pancreatic region	5 [13]	4 [11]	
Left anterior renal space	18 [47]	21 [60]	
Right anterior renal space	15 [39]	10 [29]	
Size of WON (cm^3^)	144.1 ± 20.8	156.2 ± 33.1	0.069
Inflammatory indexes			
WBC (× 10^9^/L)	16.2 ± 2.6	16.9 ± 2.8	0.753
CRP (mg/L)	126.3 ± 36.4	133.3 ± 34.7	0.403
Severity rating			
APACHE II	8.6 ± 1.9	8.4 ± 1.3	0.599
Ranson	3.9 ± 1.2	3.5 ± 0.9	0.110
CTSI	8.3 ± 1.8	8.2 ± 1.8	0.813

AP, acute pancreatitis; WON, walled-off necrosis; WBC, white blood cells; CRP, C-reactive protein; APACHE, acute physiology and chronic health evaluation; CTSI, computed tomography severity index

### Effectiveness of PCD intervention

Forty-eight hours after the PCD intervention, the CUC-PCD group demonstrated significantly lower WBC, CRP, and severity scores (APACHE II, Ranson, and CTSI) than the US-PCD group. In the CUC-PCD group, 85% of the patients showed effective imaging improvement, which was significantly higher than that in the UC-PCD group (68.42% of patients with effective imaging improvement) (Table 2).

**Table 2:** The effectiveness indicators for PCD interventions between the two groups

Variable	UC-PCD	CUC-PCD	*p* value
Average size of WON (cm^3^)	50.3 ± 7.2	44.8 ± 12.1	0.023*
Effective imaging cases (*n* [%])	26 [68.42]	30 [85.74]	0.081
Inflammatory indexes			
WBC (× 10^9^/L)	11.9 ± 1.8	10.8 ± 2.1	0.020*
CRP (mg/L)	78.1 ± 27.3	67.3 ± 12.0	0.031*
Severity rating			
APACHE II	7.4 ± 2.0	6.6 ± 1.2	0.041*
Ranson	3.1 ± 1.1	2.6 ± 0.6	0.018*
CTSI	3.3 ± 2.2	2.4 ± 1.8	0.032*

*p < 0.05

### Detailed conditions of PCD intervention

The interval between the onset of AP and PCD (*p* = 0.612), and puncture routes (*p* = 0.640) in the two groups were statistically comparable with no significant differences (Table 3). The puncture route was located in the subxiphoid (13.16% vs*.* 11.43%), left middle (26.31% vs*.* 22.86%), and posterior (25.81% vs*.* 37.14%) axillary lines, and the right middle (18.42% vs*.* 11.43%), and posterior (25.81% vs*.* 17.14%) axillary lines in the UC-PCD and CUC-PCD groups, respectively. Ten patients in the UC-PCD group received tube expansion, while only three patients in the CUC-PCD group were provided with tube expansion (*p* = 0.048). PCD catheter duration was shorter in the CUC-PCD group compared to the UC-PCD group (*p* = 0.001). Necrosectomy was more frequently performed in the CUC-PCD group compared to the UC-PCD group (*p* = 0.042).

**Table 3:** General condition of PCD interventions between the two groups

Variable	UC-PCD (*n* = 38)	CUC-PCD (*n* = 35)	*p* value
Interval between onset of AP and PCD	33.7 ± 4.7	34.3 ± 5.3	0.612
Puncture points (*n* [%])			0.640
Subxiphoid	5 [13]	4 [11]	
Left midaxillary line	10 [26]	8 [23]	
Left posterior axillary line	8 [21]	13 [37]	
Right midaxillary line	7 [18]	4 [11]	
Right posterior axillary line	8 [21]	6 [17]	
Upsizing of catheter (*n* [%])			0.048*
12–16 Fr	6 [16]	0	
16–20 Fr	3 [8]	1 [3]	
20–24 Fr	1 [3]	2 [6]	
PCD catheter duration (days)	30.2 ± 8.7	22.8 ± 8.7	0.001**
Times of necrosectomy (*n* [%])	7 [18]	14 [40]	0.042*
Once	4 [11]	12 [34]	
Multiple times	3 [8]	2 [6]	

PCD, percutaneous catheter drainage

*p < 0.05, **p < 0.01

### Operational safety of PCD

Intervention-related complications were observed in five patients in the CUC-PCD group and six patients in the UC-PCD group (*p* = 0.858). Five patients each group developed haemorrhage. One patient in the UC-PCD group experienced hollow organ damage to the colon, but no damages to the solid organs were observed in either group (Table 4). Concerning the infectious outcomes of the AP patients, there was no significant difference between the CUC-PCD and UC-PCD groups.

**Table 4:** Complications between the two groups

Variable	UC-PCD	CUC-PCD	*p* value
Intervention-related complications (*n* [%])	6 [16]	5 [14]	0.858
Haemorrhage	5 [13]	5 [14]	0.888
Parenchyma organ injury	0	0	–
Hollow visceral injury	1 [3]	0	1.000
Infection-related complications (*n* [%])			
The prevalence of bacteremia	20 [53]	18 [51]	0.918
The prevalence of sepsis	14 [37]	12 [34]	0.819

### Mortality rate and cost

The mortality rate was two of 38 patients (5.26%) in US-PCD group, and one of 35 (2.86%) in the US/CT-PCD group (*p* = 0.876). The cause of death was multiple organ failure. After PCD intervention, the CUC-PCD group showed a significantly shorter length of stay and lower hospitalisation cost than the UC-PCD group (*p* = 0.000) (Table 5). These results might be partially attributed to the greater validity of choledochoscope-assisted drainage rather than US/CT imaging fusion guidance.

**Table 5:** Treatment information between the two groups

Variable	UC-PCD	CUC-PCD	*p* value
Mortality (*n* [%])	2 [5]	1 [3]	0.876
Days in hospital after PCD	25.3 ± 4.61	18.4 ± 3.98	< 0.001***
Total cost after PCD (× 10^4^ Dollars)	0.6 ± 0.09	0.5 ± 0.06	< 0.001***

***p < 0.001

## Discussion

Based on the step-up approach for AP, the treatment strategies for liquid accumulation were summarised as “3Ds”, including Delay of surgical intervention, minimally invasive Drainage, and Debridement [14]. Among them, traditional PCD is the major intervention, which consists of image-guided puncture, drainage catheter placement, sinus dilatation via catheter enlargement, and endoscopic necrosectomy [15, 16].

Although PCD is widely used for treating infected WON, it is associated with some problems that have not yet been resolved. Inspired by endoscopic and laparoscopic drainage of pseudocysts or WONs [17], we combined the flexibility of endoscopy with the convenience of trocar establishment and fusion imaging technique to increase the current drainage efficiency. In cases with a deep location of the infected WON, the efficiency of precise drainage targeting the WON, rather than an enlarged diameter of the catheter, may determine the success of the drainage procedure [18]. Clinically efficient drainage, which can be achieved by inserting the catheter head into the sloping position in the entire cavity, might increase the risk of damage to adjacent viscera along the puncture route [19]. On the contrary, it was observed that the selected safe route, which was the straightest and shortest pathway with a particular puncture angle, generally resulted in improper placement of the drainage catheter, as the direction of the inserted guidewire could not be freely controlled. This dilemma could be effectively solved by CUC-PCD, as it facilitates precise drainage, based on the selection of the lowest point in the largest area of the WON. In this study, the laparoscopic trocar had an inner diameter of the JL5 MN type that matched the entrance of the cholangioscope and catheter (16 Fr), making the procedure possible. As a consequence of the precise placement, a smaller WON size, along with decreased inflammatory indexes and severity rating were achieved in this study. Furthermore, when necrotic tissue was observed by cholangioscopy, the mature abscess attached to the cavity wall or the divided abscess could be simultaneously debrided by cholangioscopic interventions (lavage, extraction) [20]. For achieving that, the efficacy of necrosectomy in increasing the healing process has been previously verified [17, 21, 22]. In terms of the convenience provided by the choledochoscope, necrosectomy was more frequently performed in the CUC-PCD group.

In addition, the traditional PCD period for infected WON was long due to the longer duration of sinus tract formation and dilatation via catheter expansion to ensure a sufficient drainage [11]. Only after the sinus tract (with a diameter larger than 14 Fr) was established could a choledochoscope be introduced for further interventions. Furthermore, prolonged sinus tract expansion may lead to increased complications, persistent pain, and risk of drainage failure. In the CUC-PCD group, the application of a trocar rather than a regular catheter made the drainage and necrosectomy of the WON possible in a one-stage procedure. The sheath of the punctured trocar served as a dilated mature sinus tract to prevent the spread of the infection in the abdominal cavity. Therefore, there was no need to wait for the formation and enlargement of the sinus tract, resulting in a shorter time required for expanding the sinus tract and duration of catheter placement. The drainage time used in this study was much less than that (28 days; 46 days) reported in previous studies [12, 23]. No extra infection-related complications occurred in this study.

Compared to the pigtail catheters used in traditional methods, the trocar used in CUC-PCD was larger and sharper, which can easily damage the adjacent organs along the puncture route [24]. Thus, it is imperative to determine a safe and short puncture route to reach the necrotic cavity. The safety of integrating the advantages of US and CT for PCD has been already proven [18, 25]. In addition to the imaging evolution, the efficacy of PCD depends on the route selection in terms of the different WON locations. Empirically, for lesions in the pancreatic tail region, including the spleen-kidney gap and left anterior renal space, a route from the left abdomen (left midaxillary and posterior axillary line) was recommended. Lesions adjacent to the pancreatic head (liver-kidney space, right anterior renal space) can be drained by a route along the right abdomen (right midaxillary and posterior axillary line). To drain the lesions in the pancreatic body, including the anterior part of the pancreas and the lesser omental sac area, routes through the middle and upper abdomen (subxiphoid) should be considered, although they require a more accurate assessment for safety. Using the above strategic routes, none of the patients treated by CUC-PCD experienced severe puncture complications (parenchyma or hollow viscera injury), which indicated that the imaging fusion system was also feasible for trocar puncture.

Although some patients with infected WON benefited from CUC-PCD, we acknowledge that the present study has some limitations. First, the evidence is weak because of the relatively small sample size, and patients were not randomly selected due to the retrospective nature of this study. Second, application of this technique might be currently limited to a single WON situated in deep anatomical planes, and drainage methods for multiple WONs have not yet been verified. Finally, the follow-up time was too short to reach a reliable conclusion. Based on these limitations, studies with a larger case–control prospective design, multicentre trials, and a longer follow-up time are needed to obtain more robust clinical evidence.

## Data Availability

The data are now available in your system. In detail, data (general information, blood indexes and images) that are directly related to the acquired results are available in the system submitted this time. For more detailed information, the readers can consult the first author, Hui Zhang (email: 124503329@qq.com, Telephone number: 0086-13541142792). Data concerning about admission documents are not freely available due to the patients’ privacy.

## Abbreviations

AP: Acute pancreatitis
APACHE: Acute physiology and chronic health evaluation
CT: Computer tomography
CTSI: Computed tomography severity index
CUC-PCD: Choledochoscope/US/CT/guided PCD
CRP: C-reactive protein
MSAP: Mild-severe acute pancreatitis
PCD: Percutaneous catheter drainage
SAP: Severe acute pancreatitis
US: Ultrasonography
UC-PCD: US/CT/guided PCD
WON: Walled-off necrosis
WBC: White blood cell
